# Biodesign Research to Advance the Principles and Applications of Biosystems Design

**DOI:** 10.34133/2019/9680853

**Published:** 2019-11-24

**Authors:** Xiaohan Yang, Lei S. Qi, Alfonso Jaramillo, Zong-Ming (Max) Cheng

**Affiliations:** ^1^ Biosciences Division , Oak Ridge National Laboratory , Oak Ridge , TN 37831 , USA; ^2^ The Center for Bioenergy Innovation , Oak Ridge National Laboratory , Oak Ridge , TN 37831 , USA; ^3^ Department of Bioengineering , Stanford University , Stanford , CA 94305 , USA; ^4^ Department of Chemical and Systems Biology , Stanford University , Stanford , CA 94305 , USA; ^5^ Stanford ChEM-H , Stanford University , Stanford , CA 94305 , USA; ^6^ Warwick Integrative Synthetic Biology Centre (WISB) and School of Life Sciences , University of Warwick , CV4 7AL Coventry , UK; ^7^ ISSB , CNRS , Univ Evry , CEA , Université Paris-Saclay , 91025 Evry , France; ^8^ Institute for Integrative Systems Biology (I2SysBio) , University of Valencia-CSIC , 46980 Paterna , Spain; ^9^ Department of Plant Sciences , University of Tennessee , Knoxville , TN 37996 , USA; ^10^ Nanjing Agricultural University , Nanjing , Jiangsu Province , China

Over the course of civilization, humans have increasingly expanded their freedom to live a better life. In comparison with the primitive society, our modern society has many more choices of life-supporting resources, such as year-round food supply, permanent shelters, diverse energy sources, and effective preventive and curing medicine. However, our society is currently still heavily relying on the resources provided by Mother Nature, which cannot meet the future global needs in terms of both quantity and quality under the pressure of population growth, natural resource reduction, and environmental deterioration. For example, the food sources originating from plants, animals, or microbes do not have the nutrition balance for optimal human health [ 1 – 3 ]. Climate change and environmental deterioration threaten the food security [ 4 – 6 ]. Increasingly, infectious diseases (e.g., HIV/AIDS), genetic diseases (e.g., cancer), and improper lifestyle-related disorders (e.g., obesity) become more prevalent and remain challenging to be prevented, controlled, and cured. Conventional medical technologies and modern medicine development are also meeting the ceiling. Antibiotic resistance is threatening the health of humans, animals, and environment [ 7 ]. As the human lifespan continues to increase, aging-related diseases, disorders, and poor life quality are becoming global challenges [ 8 ]. Plants, animals, and microbes in nature have evolved as part of our Earth ecosystem, not for benefiting humans. Even though humans have put forth tremendous efforts to domesticate plants, animals, and microbes based on random/induced mutations, hybridization, and limited genetic modifications via biological engineering, the improved food and industrial crop plants, animals, and microbial strains are still far from optimized for meeting the human needs. In other words, natural evolution and domestication in plants, animals, and microbes are tinkering processes and therefore cannot meet the ever-exploding population on the one hand and the unending appetite for living better quality life on the other. One promising strategy for solving these global challenges is to employ revolutionary biosystems design (also called biodesign), which is defined as predictable modification of existing organisms or creation of new organisms using rational engineering or automated design based on the theory and principles of biosystems design.

**Figure 1:**
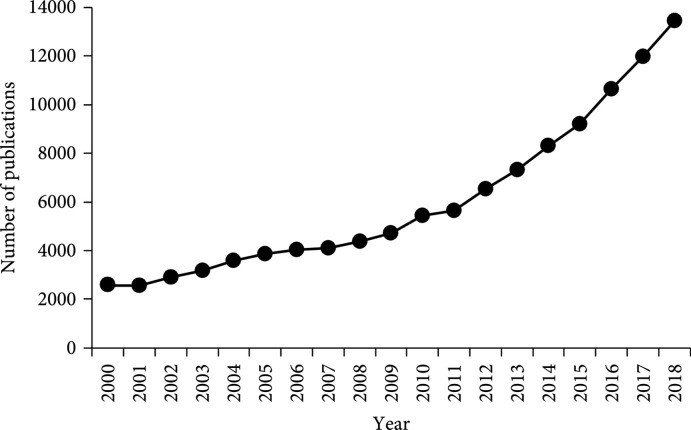
Number of biodesign-related publications in PubMed per year during 2000–2018. The search term is “artificial cell[tw] OR base editing[tw] OR biodesign[tw] OR bioengineering[tw] OR biological engineering[tw] OR biosystems design[tw] OR CRISPR[tw] OR gene circuit[tw] OR gene editing[tw] OR gene therapy[tw] OR genetic circuit[tw] OR genome editing[tw] OR genome writing[tw] OR metabolic engineering[tw] OR metabolic modeling[tw] OR pathway engineering[tw] OR pathway modeling[tw] OR synthetic biology[tw] OR tissue engineering[tw]”.

Biodesign in living organisms deals with the structure of the particular organism and the interactions between the biomolecules encoded in their genomes. In the broadest sense, biodesign is concerned with the nature and properties of the information required to create biological behavior at the ecological, organism, cellular, organelle, and molecular levels and how biological systems interface with the environment and devices. The explosion of omics data (i.e., genomics, transcriptomics, proteomics, metabolomics, and phenomics), the rapid advance in computational modeling and machine learning, the reduced cost of omics data generation and analysis and DNA/RNA and protein synthesis, and the disruptive genome editing technology based on CRISPR- (Clustered Regularly Interspaced Short Palindromic Repeats-) Cas (CRISPR-associated) systems have accelerated the evolution of biodesign as a next-generation discipline of biology, as best reflected by a rapid increase in the number of publications related to biodesign since a decade ago (Figure 1 ). As a consequence of the new paradigm shift of biology by integrating genome reading with genome editing and rewriting, biodesign promises to solve three critical challenges: (1) integrating large-scale omics data with computational modeling to gain a comprehensive and deep understanding of the structure and function of complex networks and pathways in all types of organisms and to systematically link genes (causes) to traits (effects or phenotypes); (2) improving the existing commodity organisms (e.g., agricultural and industrial crops, livestock, and industrial microbes) and most importantly human health through molecular design of new pharmaceuticals, postembryotic gene editing and therapy, intelligent genetic circuits, and pathway/tissue engineering; and (3) creating novel traits or function through large-scale DNA synthesis and genome writing and rewriting within the framework of social responsibility and professional ethics.

With unlimited potential in fundamental research, broad practical applications, and ever-increasing amount of R&D funding support from government agencies, industries, and government/industry consortia around the world, biodesign is growing into a new interdisciplinary field that integrates a wide range of research areas, including molecular biology, genetics, biochemistry, genomics, chemical engineering, computational biology, bioinformatics, synthetic biology, systems biology, physiology, ecology, breeding, medical science, animal science, microbiology, crop science, horticulture, and forestry. To embrace the promise of this fast-growing research discipline, this journal was launched to advance both fundamental and applied research, foster multidisciplinary collaboration, and promote dissemination of scientific information and knowledge in the field of biosystems design. This new journal will emphasize the rational or automated design of engineered organisms to address global challenges in health, agriculture, bioenergy, and the environment. It will promote research, with the increasing sophistication and greater social responsibility, on the predictable conduct of (1) engineered biological parts in an exogenous genomic context, (2) engineered part assemblies (gene circuits, pathways, and macromolecular assemblies) in an exogenous compartment context, (3) engineered assemblies in an exogenous cell, (4) engineered cells in an exogenous tissue, and (5) engineered tissues in an exogenous organism.

**Figure 2:**
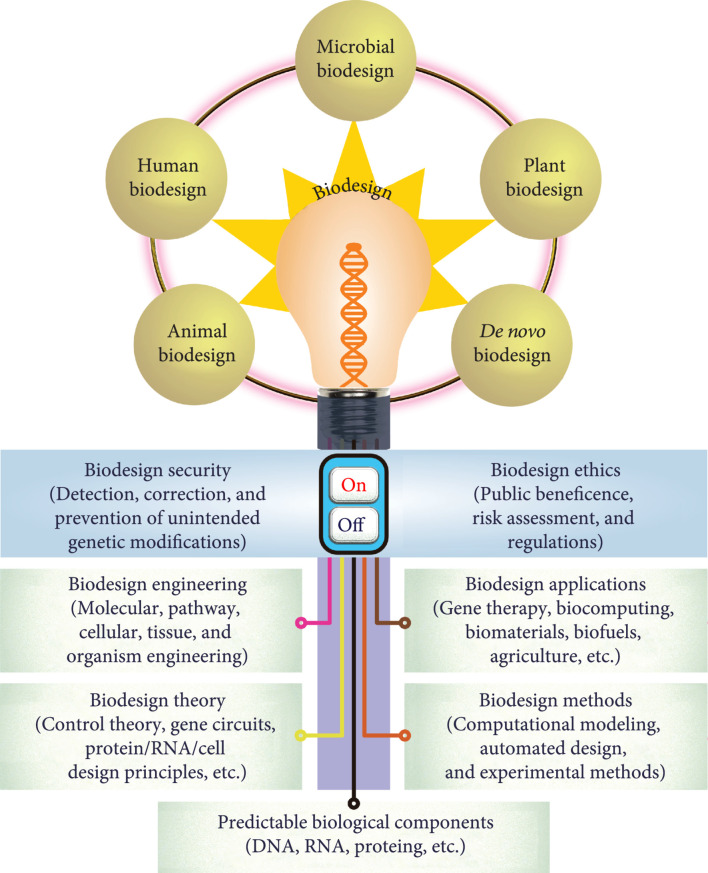
This journal features a multidimensional framework integrating scientific dimension, organismal dimension, and social responsibility dimension. The scientific dimension includes biodesign theory, methods, engineering, and applications; the organismal dimension includes animal, human, microbial, plant, and *de novo* organism biodesign; and the social responsibility dimension includes biodesign security and ethics. As a double-edged sword, the biosystems design research should be regulated by an “on/off” switch based on the aspects of biodesign security and ethics.

This journal will feature a framework that integrates the following three dimensions: (1) scientific dimension: biodesign theory, principles, methodology, tools, and applications; (2) organismal dimension: animal, human, microbial, plant, and *de novo* organism biodesign; and (3) social responsibility dimension: biodesign security and ethics (Figure 2 ). While biosystems design research can make a huge positive impact on a wide range of disciplines, it should be regulated by an “on/off” switch based on the aspects of biodesign security and ethics to avoid any negative consequences, such as the unethical use of gene editing in human germ cells [ 9 , 10 ].

As a vigorously peer-reviewed, open-access online-only journal, it is dedicated to rapid, efficient, and free worldwide dissemination of scientific discoveries, technological advancements, and social responsibilities in the format of research articles, reviews, editorials, and perspectives. We are honored and humbled to serve the biodesign community as the founding editors-in-chief and executive editor and are extremely grateful to highly reputable experts to serve as associate editors. This journal welcomes high-quality manuscripts from researchers around the world. We hope that this journal will grow fast and healthy to become a leading journal in the field of biodesign through collective efforts of the editorial board, authors, reviewers, and readers.
